# Evidence of exposure and human seroconversion during an outbreak of avian influenza A(H5N1) among poultry in Cameroon

**DOI:** 10.1080/22221751.2018.1564631

**Published:** 2019-01-22

**Authors:** Chavely Gwladys Monamele, Phalla Y., Erik Albert Karlsson, Marie-Astrid Vernet, Abel Wade, Marie-Claire Assoumou Okomo, Aristide Stéphane Abah Abah, Sokhoun Yann, Georges Alain Mballa Etoundi, Njankouo Ripa Mohamadou, Jean-Marc Feussom, Sreyviseth Horm, Paul Francis Horwood, Sowath Ly, Richard Njouom, Philippe Dussart

**Affiliations:** a Virology Department, Centre Pasteur du Cameroun, Institut Pasteur International Network, Yaoundé, Cameroon; b Virology Unit, Institut Pasteur du Cambodge, Institut Pasteur International Network, Phnom Penh, Cambodia; c Ministry of Livestock, Fisheries and Animal Industries, Yaoundé, Cameroon; d National Laboratory of Public Health, Yaoundé, Cameroon; e Ministry of Public Health, Yaoundé, Cameroon; f Australian Institute of Tropical Health and Medicine, James Cook University, Cairns, Australia; g Epidemiology and Public Health Unit, Institut Pasteur du Cambodge, Institut Pasteur International Network, Phnom Penh, Cambodia

**Keywords:** Avian influenza, H5N1, Africa, Cameroon, Zoonoses, surveillance, outbreak

## Abstract

From May 2016 to March 2017, 22 poultry outbreaks of avian influenza A(H5N1) were reported in Cameroon, mainly in poultry farms and live bird markets. No human cases were reported. In this study, we sought to describe the 2016 A(H5N1) outbreak strain and to investigate the risk of infection in exposed individuals. We find that highly pathogenic influenza subtype A(H5N1), clade 2.3.2.1c from Cameroon is closely related phylogenetically and antigenically to strains isolated in central and western Africa at the time. No molecular markers of increased human transmissibility were noted; however, seroconversion was detected in two poultry workers (1.5% of total screened). Therefore, the continued outbreaks of avian influenza in poultry and the risk of zoonotic human infection highlight the crucial need for continued and vigilant influenza surveillance and research in Africa, especially in areas of high poultry trade, such as Cameroon.

## Introduction

Highly pathogenic avian influenza virus (HPAIV) presents a major concern to the poultry industry. HPAI A(H5N1) viruses have caused thousands of outbreaks in poultry worldwide, killing tens of millions of birds and necessitating the culling of hundreds of millions more [1,2]. Since first emerging in Asia in 1996, the Eurasian lineage of influenza A(H5N1) has become enzootic, especially in Southeast Asia, and has spread globally throughout Asia, Europe, Africa, and North America [2,3]. Although sustained human-to-human transmission has not been reported, HPAI A(H5N1) viruses remain a significant public health concern and it may be only a matter of time before the virus becomes transmissible in humans [4,5]. To date, 856 human cases of A(H5N1) infection have been reported with 454 deaths (case fatality rate: 53%) [6]. The majority of human cases are associated with exposure to infected poultry at live bird markets (LBMs) or backyard farms [7].

HPAI A(H5N1) viruses have been detected in African countries since 2006. The first outbreak was recorded in Kaduna State, Nigeria, in mid-January 2006. Less than a month later, the virus was detected in Egypt, Niger, and Cameroon [8]. Since that time, further HPAIV outbreaks have been recorded in Burkina Faso, Democratic Republic of Congo, Cameroon, Côte d’Ivoire, Ghana, Niger, Nigeria, Togo, South Africa, Uganda, and Zimbabwe [9]. In West Africa, HPAIV outbreaks occurring between 2006 and 2008 were exclusively caused by clade 2.2 viruses [10]. Following a 6-year disappearance, HPAIV A(H5N1) was detected in an LBM in Lagos State in southwestern Nigeria in December of 2014; however, this virus belonged to clade 2.3.2.1c. This HPAIV quickly spread throughout West Africa to Burkina Faso, Côte d’Ivoire, Ghana, and Niger during 2015 then appeared in Central Africa in 2016 [9,11]. No human cases have been reported to date.

Cameroon is a Central African country made up of 10 administrative regions. Yaoundé, the capital city, is located in the Centre region of the country and is the second largest city in Cameroon. After Nigeria, Cameroon is the second highest poultry producer in West and Central Africa. LBMs are common in Cameroon and represent the major source of domestic poultry commerce while poultry farms supply the industrial and commercial industries. Therefore, while the poultry industry represents a flourishing economic sector in Cameroon, rates of occupational exposure to avian influenza virus remain unknown [12].

In Cameroon, three outbreaks of HPAIV A(H5N1) occurred between 21 February and 16 March in 2006 in ducks in three different localities in the northern part of the country and caused more than a hundred poultry deaths. In response, the government of Cameroon set up a biosafety zone in order to contain the virus [13]. In May 2016, following an 8-year absence, Yaoundé experienced another outbreak of HPAIV A(H5N1) in a poultry farm with approximately 15,000 bird deaths (45.5% of birds in the facility). Following this outbreak, 21 additional outbreaks of HPAIV A(H5N1) were reported between May 2016 and March 2017 in four regions of Cameroon (Centre, South, West, and Adamawa) resulting in 24,668 poultry deaths and the culling of more than 76,000 birds [14]. No human cases were reported. In this study, we sought to describe the 2016 HPAIV A(H5N1) outbreak strain and to investigate the risk of subclinical human infections with avian influenza in exposed poultry workers.

## Results

### Prevalence and detection of influenza A(H5N1) in poultry in Cameroon

**Figure 1.**
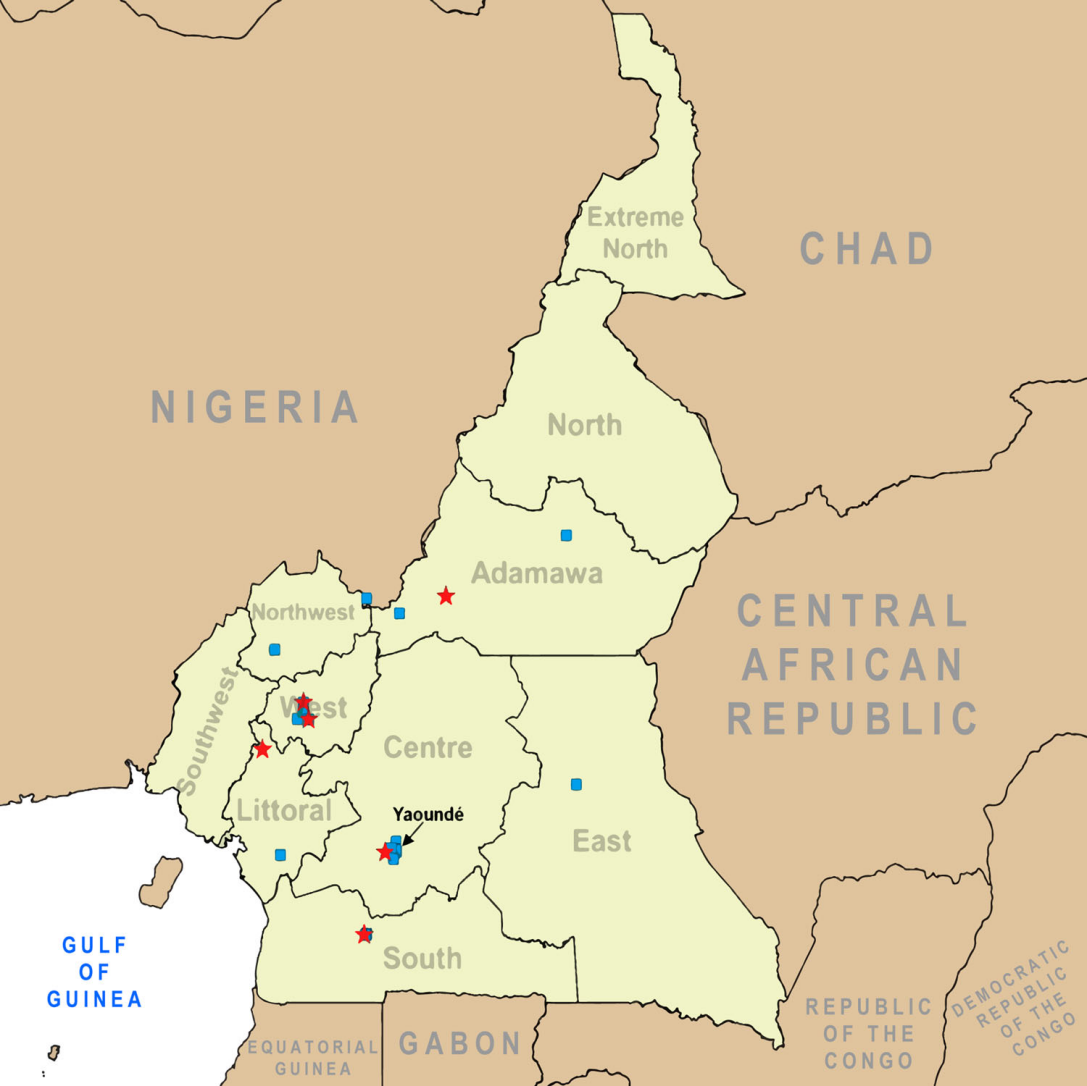
Map of poultry and human sampling sites in Cameroon. Sites where sampling took place for avian influenza prevalence and exposure are indicated on the map of Cameroon in central Africa. Blue squares represent poultry sampling locations. Red stars indicate human sampling locations.

Overall, 147 poultry samples from 7 regions in 19 total sites (Figure 1; Supplemental Table 1) were sent to the Centre Pasteur du Cameroon (CPC) for analyses between 24 May and 11 June 2016. Total prevalence of confirmed influenza A by RT-qPCR analysis was 39.5% (*n* = 58) with 34 of these samples (58.6% of influenza positive birds) confirmed as HPAIV A(H5N1) (Table 1).

**Table 1.** Number and percentage of influenza A(H5N1) positive samples identified in poultry in Cameroon between May and June 2016.

		Number of samples	Influenza A *n* (% total)	A(H5N1) *n* (% flu positive)
Time period	May 2016	56	13 (23.2)	12 (92.3)
	June 2016	91	31 (34.1)	22 (71.0)
Region	Adamawa	27	n.d.	n.d.
	Centre	59	19 (32.2)	15 (78.9)
	East	8	n.d.	n.d.
	North West	3	1 (33.3)	n.d.
	South	24	11 (45.8)	6 (54.5)
	West	26	13 (50)	13 (100)
Total		147	58 (39.5)	34 (58.6)

Note: n.d.: not detected.

### A(H5N1) viruses from Cameroon in 2016 are of the clade 2.3.2.1c

**Figure 2.**
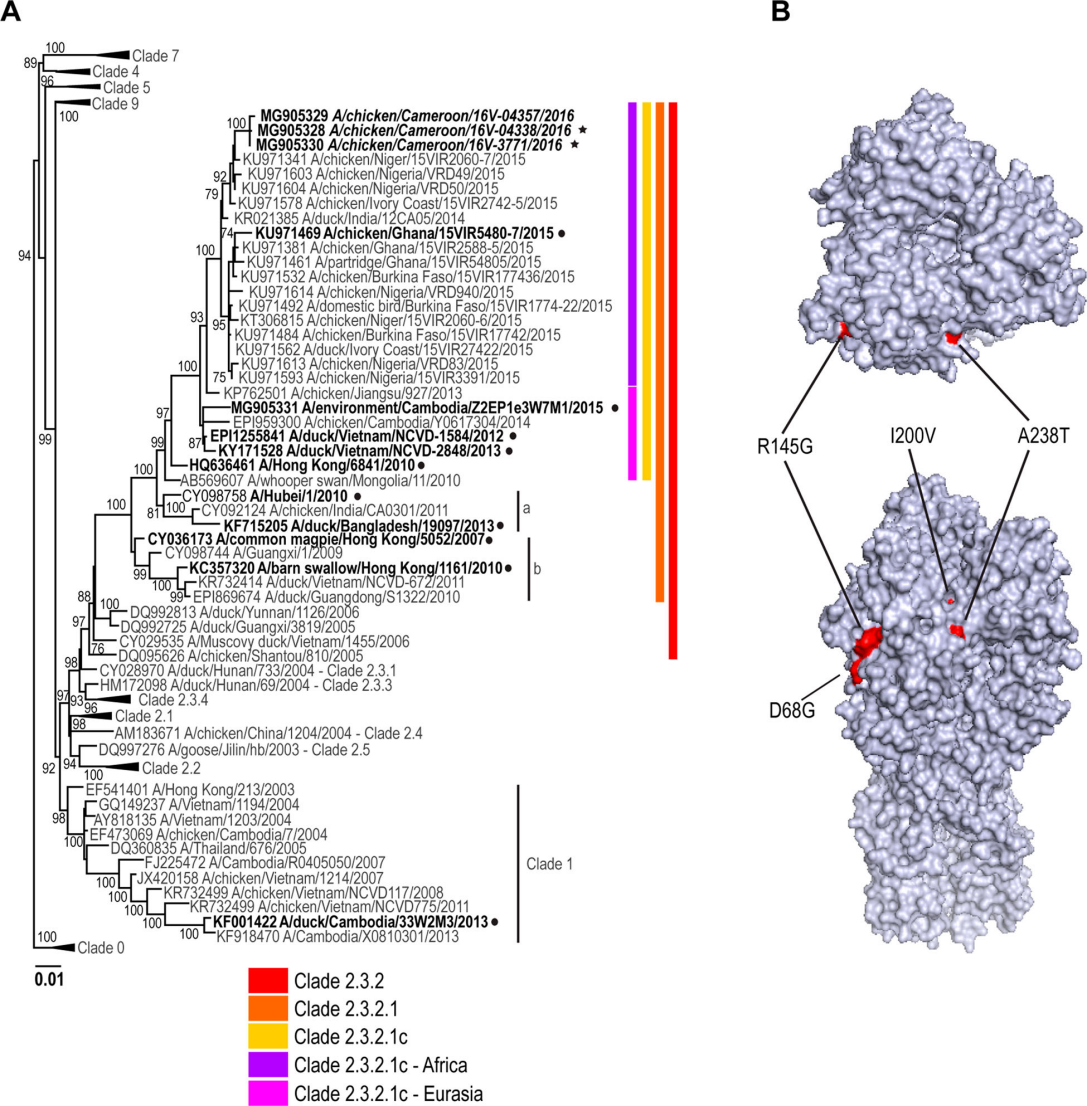
Phylogenetic analysis of the A(H5N1) haemagglutinin (HA) genes from representative viruses from Cameroon (**A**). The phylogenetic tree was generated using the maximum-likelihood method. Bootstrap values (*n* = 500) > 70 are shown. Scale bars indicate substitutions per site. Sequences from representative Cameroonian strains included in the study are indicated in bold and italic. Other control strains are indicated in bold. Representative Cameroonian isolates used in the study for antigenic analysis are indicated with black stars while control strains are indicated with black circles. Amino acid differences determined in representative strains as compared to other clade 2.3.2.1c HA genes are indicated in red on the model (**B**). Positions are stated under H5 numbering.

**Figure 3.**
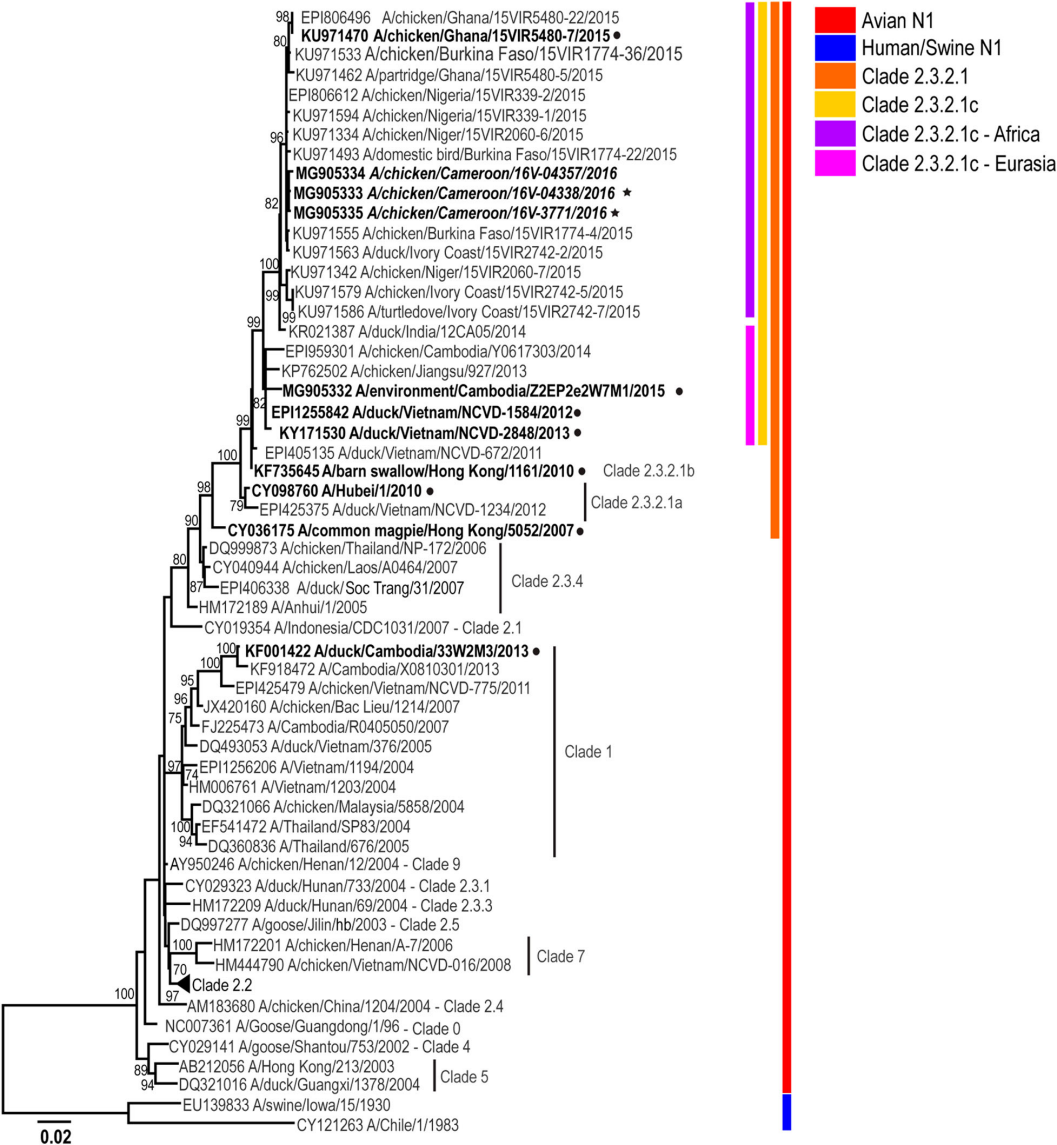
Phylogenetic analysis of the A(H5N1) neuraminidase (NA) genes from representative viruses from Cameroon. The phylogenetic tree was generated using the maximum-likelihood method. Bootstrap values (*n* = 500) > 70 are shown. Scale bars indicate substitutions per site. Sequences from representative Cameroonian strains included in the study are indicated in bold and italic. Other control strains are indicated in bold. Representative Cameroonian isolates used for antigenic analysis are indicated with black stars while control strains are indicated with black circles.

Topology of the phylogenetic trees for the haemagglutinin (HA) gene of representative isolates of HPAIV A(H5N1) viruses from Cameroon indicates that these viruses fall into genetic clade 2.3.2.1c (Figures 2 and 3; Supplemental Figure 1). All genes from representative viruses were closely related to sequences from West Africa [15,16] (99% sequence identity, Supplemental Table 4) as well as with the sequences from Europe and Asia suggesting a common virus progenitor. As previously reported [16], the PB2 gene segment of the representative Cameroon isolates appears to be a result of genetic reassortment between A(H5N1) and A(H9N2), similar to other viruses circulating in African and Asian countries [15,16].

### Molecular characterization of avian viruses

As expected, all of the representative Cameroonian A(H5N1) viruses possessed molecular markers characteristic of HPAI viruses including a multibasic amino acid cleavage site motif, PQRERRRKR*G. Interestingly, amino acid comparison of the HA gene of the Cameroonian isolates from this study and other 2.3.2.1c strains from West Africa, Europe, and China revealed two major mutations: I200V and A238T (H5 numbering, Table 2, Figure 2). No other known specific mutations, such as at positions 222 or 224 [17] were detected in the HA protein. Representative isolates also contain a deletion of the NA stalk motif known to increase pathogenicity in mice and chickens [18]. No gene mutations associated with antiviral resistance [19] were detected in any strain analysed in this study. Mutations associated with mammalian adaptation in the PB2 gene, namely E627K and D701N [20], were not detected.

**Table 2.** Comparison of amino acids in the HA gene from representative Cameroonian strains used in this study and other 2.3.2.1c strains.

Protein	Position^a^	H5N1 Cameroon	West African strains	Europe	China
HA	68	G	G	G	S/D
	145	G	R (Niger, G)	R	R
	200	V	I	I	I
	238	T	A	A	A

^a^H5 numbering.

### Antigenic testing of Cameroon isolates

Representative viral isolates from Cameroon showed highest cross-reaction against 2.3.2.1c virus antibodies, especially against the 2015 strain from Ghana (A/chicken/Ghana/7/2015). As shown in previous studies [21], strongest reactivity was noted with horse and human type O red blood cells (RBCs; Table 3).

**Table 3.** Antigenic analysis of representative A(H5N1) isolates from Cameroon.

Reference antiserum																					
		A/common magpie/Hong Kong/5052/2007				A/Hong Kong/6841/2010				A/Hubei/1/2010				A/duck/Bangladesh/19097/2013				A/barm swallow/HK/1161/2010			
	**RBC**	c	t	ho	hu	c	t	ho	hu	c	t	ho	hu	c	T	ho	hu	c	t	ho	hu
Reference virus/antigen	**Clade**																				
A/common magpie/Hong Kong/5052/2007	2.3.2.1	**320**	**320**	**2560**	**1280**	320	320	2560	1280	20	20	320	80	80	160	1280	320	40	40	0	160
A/Hong Kong/6841/2010	2.3.2.1	80	40	N/A	320	**320**	**160**	N/A	**1280**	20	20	N/A	80	320	160	N/A	640	20	20	N/A	80
A/Hubei/1/2010	2.3.2.1a	320	320	1280	160	640	640	2560	640	**320**	**320**	**1280**	**320**	640	1280	2560	640	40	80	0	160
A/duck/Bangladesh/19097/2013	2.3.2.1a	160	160	1280	320	320	640	2560	1280	20	40	320	80	**320**	**640**	**2560**	**1280**	20	40	10	80
A/barm swallow/HK/1161/2010	2.3.2.1b	80	80	640	320	160	320	1280	1280	10	20	160	80	80	160	1280	640	**40**	**40**	**0**	**320**
A/duck/VN/NCVD-1584/2012	2.3.2.1c	160	160	N/A	640	640	640	N/A	2560	40	40	N/A	160	640	640	N/A	1280	40	80	N/A	160
A/duck Cambodia/33W2M3/2013	1.1	10	0	80	20	160	80	320	320	20	10	80	80	10	0	40	20	0	0	0	10
A/envir/Cambodia/z2EP1e3W7M1/2015	2.3.2.1c	160	0	2560	10	320	0	2560	10	20	0	320	40	40	10	640	20	20	0	0	20
A/chicken/Cameroon/16V-04338/2015	2.3.2.1c	10	20	320	80	40	80	640	320	0	0	80	20	20	40	160	40	0	0	0	20
A/chicken/Cameroon/16V-3771/2015	2.3.2.1c	40	40	640	160	160	160	1280	640	10	0	160	20	40	80	640	160	10	0	0	20
		A/duck/VN/NCVD-1584/2012				A/duck/VN/NCVD-2848/2013				A/duck Cambodia/33W2M3/2013				A/chicken/Guiyang/1153/2016				A/chicken/Ghana/7/2015			
	**RBC**	c	t	ho	hu	c	t	ho	hu	c	t	ho	hu	c	t	ho	hu	c	t	ho	hu
Reference virus/antigen	**Clade**																				
A/common magpie/Hong Kong/5052/2007	2.3.2.1	80	80	1280	320	80	160	0	320	0	20	0	20	0	0	160	10	80	80	1280	320
A/Hong Kong/6841/2010	2.3.2.1	320	160	N/A	1280	80	80	N/A	640	0	0	N/A	40	0	0	N/A	20	80	40	N/A	640
A/Hubei/1/2010	2.3.2.1a	160	320	2560	320	80	160	0	640	40	40	0	80	0	10	80	20	80	160	1280	320
A/duck/Bangladesh/19097/2013	2.3.2.1a	160	640	2560	1280	80	160	10	320	0	10	10	20	0	10	10	10	40	80	1280	320
A/barm swallow/HK/1161/2010	2.3.2.1b	80	80	1280	640	40	80	0	320	0	10	0	20	0	10	0	10	40	40	320	160
A/duck/VN/NCVD-1584/2012	2.3.2.1c	**640**	**640**	**N/A**	**2560**	160	160	N/A	1280	10	10	N/A	40	10	0	N/A	20	160	160	N/A	640
A/duck Cambodia/33W2M3/2013	1.1	20	0	80	40	10	0	0	20	**640**	**160**	**0**	**1280**	0	0	0	20	80	40	320	160
A/envir/Cambodia/z2EP1e3W7M1/2015	2.3.2.1c	80	10	640	20	80	80	0	320	0	0	0	20	0	0	160	10	40	0	1280	10
A/chicken/Cameroon/16V-04338/2015	2.3.2.1c	20	20	320	80	10	10	0	40	10	10	0	40	0	0	80	0	80	160	1280	320
A/chicken/Cameroon/16V-3771/2015	2.3.2.1c	40	80	640	160	10	20	0	80	10	20	0	40	0	0	160	10	160	320	2560	640

Note: RBC: red blood cells; c: chicken; t: turkey; ho: horse; hu: human; N/A: not available.

### No detection of avian influenza infection in nasaopharyngeal or oral swabs from poultry workers in Cameroon

A total of 663 subjects were sampled for exposure to avian influenza based on presence on a farm or in a market where dead or dying poultry was identified. Swab samples (pooled NP and OP samples) were collected from 5 regions encompassing 7 total sites (Figure 1; Supplemental Table 1) during the outbreak period between May and July 2016. Overall, the study population age ranged from 6 months to 80 years with a mean age of 31 years. The majority of the potentially exposed population was male with a male/female sex ratio of 3:1. No samples were positive for avian influenza (subtypes H5 and H7) by RT-qPCR; however, 2.3% (15/663) of the samples were positive for influenza A virus, 73.3% (11/15) of which were positive for the human seasonal A(H3N2) subtype (Table 4).

**Table 4.** Socio-demographic characteristics and influenza detection in exposed poultry workers and close contacts in Cameroon between May and July 2016.

		Number tested	Number Positive n (% total)	A/pdmH1N1 *n* (% flu positive)	A/H3N2 *n* (% flu positive)	Untyped *n* (% flu positive)	A(H5N1) *n* (% flu positive)	A/H7N9 *n* (% flu positive)
Sex	Male	481	12 (2.5)	n.d.	9 (75.0)	3 (25)	n.d.	n.d.
	Female	157	3 (1.9)	n.d.	2 (66.6)	1 (33.3)	n.d.	n.d.
	n/a	25	n.d.	n.d.	n.d.	n.d.	n.d.	n.d.
Age	0-20.	130	1 (0.8)	n.d.	n.d.	1 (100)	n.d.	n.d.
	21–40	385	11 (2.9)	n.d.	9 (81.8)	2 (18.2)	n.d.	n.d.
	41–60	118	2 (1.7)	n.d.	1 (50)	1 (50)	n.d.	n.d.
	61–80	16	1 (6.3)	n.d.	1 (100)	n.d.	n.d.	n.d.
	n/a	14	n.d.	n.d.	n.d.	n.d.	n.d.	n.d.
Region	Adamawa	97	n.d.	n.d.	n.d.	n.d.	n.d.	n.d.
	Centre	232	7 (3.0)	n.d.	6 (85.7)	1 (14.3)	n.d.	n.d.
	Littoral	33	n.d.	n.d.	n.d.	n.d.	n.d.	n.d.
	South	149	4 (2.7)	n.d.	3 (75)	1 (25)	n.d.	n.d.
	West	152	4 (2.6)	n.d.	2 (50)	2 (50)	n.d.	n.d.
Total		663	15 (2.3%)	n.d.	11 (73.3)	4 (26.7)	n.d.	n.d.

Note: n.d.: not detected; n/a: not available.

### Prior exposure and seroconversion in a longitudinal human serosurvey for A(H5N1) infection in poultry workers from Cameroon

Despite not being able to detect any active avian influenza infections in poultry workers, longitudinal serosurvey suggested both prior exposure and seroconversion against Cameroonian strains of HPAIV A(H5N1) among poultry farm and LBM workers exposed to diseased or dead poultry. Of the 131 participants selected for serological analysis, 16 (12.2%) had the possible presence of antibodies against A(H5N1) isolates from Cameroon with a reciprocal HAI titre ≥ 10 on the second sampling. Three (2.3%) of these individuals had reciprocal HAI titre ≥ 20. Of the individuals positive in the second sampling, 2 (1.5%) were found to have a 4-fold increase in HAI titre between the first and second serum collections, suggesting seroconversion. Interestingly, some poultry farm and LBM workers showed an increased seroprevalence against the control Cambodian 2.3.2.1c virus strain (A/environment/Cambodia/z2EP1e3W7M1/2015) used for testing; however, it is unclear whether this could be from prior exposure or cross-reactivity due to the lack of cognate antiserum against this viral strain for analysis (Supplemental Table 6).

## Discussion

Overall, these studies utilizing representative isolates indicate that HPAI A(H5N1) viruses circulating in Cameroon in 2016 were of the clade 2.3.2.1c and were closely related to other outbreak strains isolated during the same period across West and Central Africa. While it is unclear exactly how A(H5N1) was introduced into Cameroon, it is hypothesized that migration of infected waterfowl and/or trade of domestic poultry with neighbouring countries could serve as possible sources. Since Nigeria was the first country in Africa to report HPAIV A(H5N1) [16], it is hypothesized that all A(H5N1) strains in West and Central Africa from this outbreak period likely originated from a Nigerian source. These strains may have been introduced into Africa from Asia, where there were reports of hundreds of migratory birds found dead in January 2015 due to exposure to a novel, reassortant HPAIV possessing a clade 2.3.2.1c HA gene and an H9N2-derived PB2 gene [22]. Aside from direct introduction from Asia into Nigeria [16], these viruses could also have been introduced into West and, subsequently, Central Africa through the westward spread of clade 2.3.2.1c viruses on a global scale from unidentified Asian sources [23]. Indeed, 2.3.2.1c viruses isolated in Dubai in 2014 are in the direct ancestry of viruses detected in West Africa, suggesting a possible stepping-stone. However, a direct pathway of spread into Nigeria remains unclear. Further sequencing and analysis of larger data sets than the representative isolates included in this study are warranted to better determine the viral origin.

While no active AIV infections were detected in poultry farm and LBM workers, serological results do suggest exposure to avian influenza viruses in these high-risk groups, likely through contact with diseased or dead poultry. No poultry workers reported any symptoms in relation to acute HPAIV infection. Therefore, seroconversion determined in this study could be related to subclinical or very mild cases. This assumption is not unreasonable as clade 2.3.2.1c viruses appear to have minimal morbidity or mortality in humans, as exemplified by the drop in number of cases observed in Cambodia following the A(H5N1) clade replacement from 1.1.2–2.3.2.1c in 2014 [24]. It is currently unclear why these 2.3.2.1c viruses are not associated with severe avian influenza infection in humans. The A238T mutation observed in the HA of the representative viruses included in this study has been shown previously to add a glycolysation site, ^236^NDT, potentially altering infectivity or antigenicity; however, the exact contribution of this site is unknown [25]. Further, the contributions of the other HA mutations observed in these representative viruses have not yet been studied. In addition, mutations E627K and D701N in the PB2 gene that have been reported to facilitate the adaptation of avian viruses to mammals, and increase transmission and/or pathogenicity [20,26,27] were not observed in the Cameroonian isolates analysed and could be one contributing factor to the lack of severe human cases reported in these outbreaks.

Previous reports suggest that asymptomatic and/or mild avian influenza infections lead to seroconversion with low antibody titres that quickly decrease below the World Health Organization (WHO) confirmation threshold (reciprocal HAI ≥ 160; MN ≥ 80) [28]. For this reason, we considered as positive all individuals with a reciprocal HAI titre ≥ 40 and a reciprocal MN titre ≥ 40, which is less stringent than the WHO cut-off levels. However, no current consensus exists on the antibody titre that results from a mild or asymptomatic infection, and cut-off values used in previous studies are inconsistent. Therefore, taking into consideration the antigenic analysis, multiple antigen testing, increases in titres between samplings, and confirmation by microneutralization testing, the observed seroconversion in 2 out of the 131 participants was considered positive. Overall, 2.3% of workers who participated in the study had antibodies against A(H5N1) (either pre-existing antibodies or seroconversion). While this prevalence is lower than reported among LBM workers in Cambodia [29], Thailand [30], Nigeria [31], or China [32], it is higher than in studies of poultry workers in Nigeria [33] and residents of villages with human cases in Thailand [34].

While Cameroon has not experienced an outbreak of HPAIV A(H5N1) since March of 2017, avian influenza remains a threat to West and Central Africa [9]. For many years, the prevalence of avian influenza in West and Central Africa was thought to be relatively low [12,35]; however, recently, avian influenza clade 2.3.4.4 (A/H5N8) has been detected in West and Central Africa, including Cameroon, and these viruses are still being detected in Nigeria as recently as September of 2017 [9]. While thought to have low pathogenicity and transmissibility in humans [36–38], the spread of these viruses to Africa highlights the continual risk of incursion and circulation of new strains. In addition, with the booming poultry farming industry in the region, occupational exposure to domestic poultry in West and Central Africa, including Cameroon, is estimated to be high [12,39]. Therefore, continued outbreaks and detection of avian influenza in poultry coupled with the clear and present risk of zoonotic human infection highlight the crucial need for continued and vigilant influenza surveillance and research in Africa, especially in areas of high poultry trade such as the LBMs and poultry farms of Cameroon.

## Materials and methods

### Avian samples

#### Ethical approval

The animal investigation was conducted as part of the outbreak response in Cameroon at the request of the Cameroonian veterinary authorities to help in assessing and eradicating the outbreak; thus, it was not considered to be experimental animal research. CPC serves as the National Influenza Centre for the country. Administrative authorizations were obtained from the Cameroon Ministry of Health in order to control the spread of infection. Export of samples from Cameroon into Cambodia followed strict international regulations (including IATA shipping regulations) as well as specific regulations according to each individual country. Institute Pasteur in Cambodia serves as the WHO Regional H5 Reference Laboratory as well as the National Influenza Center of Cambodia and has all approvals and capabilities necessary to work on highly pathogenic avian influenza. No animal experimentation was performed at IPC.

#### Sample collection

During the outbreak period, poultry samples were collected from 7 regions in 19 total sites (Supplemental Table 1) and sent to Centre Pasteur du Cameroun (CPC) as well as the National Veterinary Laboratory where all analyses were performed and confirmed. Tracheal and cloacal swabs were collected in addition to post-mortem organ biopsies for dead poultry [40]. All samples were placed in 2 mL viral transport medium before transportation to the CPC on ice to maintain cold chain.

#### Nucleic acid extraction and amplification

All samples were processed under BSL3 containment conditions [41]. Nucleic acids were extracted from the samples using the QIAamp Viral RNA Mini Kit (Qiagen, Hilden, Germany) according to the manufacturer’s instructions. RNA extracts were tested by quantitative real-time reverse-transcriptase polymerase chain reaction (RT-qPCR) on an ABI Prism 7300 or 7500 thermocycler (Applied Biosystems, Foster City, California, USA) for the detection of influenza A as well as H5 and H7 subtypes with the CDC Influenza typing and sub-typing assay obtained through the International Reagent Resource Program (IRR, https://www.internationalreagentresource.org). The typing and sub-typing assay protocols were conducted with the enzyme Ambion AgPath-ID^™^ One-Step RT–PCR Kit (ThermoFisher Scientific, Massachusetts, USA). A reaction volume of 25 µL contained 12.5 µL of 2× reaction mix, 1 µL water, 0.5 µL enzyme, 2 µL of 10 µM forward primer, 2 µL of 10 µM reverse primer, 2 µL of 2.5 µM probe and 5 µL RNA extract. The following program was used for amplification: 30 min at 50°C, 10 min at 95°C, 40 cycles of 15 s at 95°C and 30 s at 55°C. RT-qPCR results with Ct values below 37 were considered positive.

Samples positive for H5 genes were further analysed for the presence of N1 gene with the following set of primers: forward A/N1/+/2 (5′-GCAAAAGCAGGAGTTTAAAATGAA-3′), and reverse A/N1/-/1431 (5′-ACTTGTCAATGGTGAATGGCAAC-3′) [13].

#### Egg isolation and sequencing

To achieve the objectives of characterizing and identifying the HPAIV virus associated with the Cameroonian outbreak, and to have isolates available for serological testing, three representative samples positive for the H5 HA gene were selected for isolation and sequencing at the Pasteur Institute of Cambodia (IPC). Samples were selected based on Ct value (<21), availability of sufficient sample, and geographical origin. Specifically, selected samples represent three different outbreaks in the Centre and the West regions of Cameroon. Avian samples were inoculated into specific pathogen-free, 10 days old embryonated chicken eggs according to previously published protocols [42]. Two viral isolates were successfully recovered following egg inoculation: A/chicken/Cameroon/16V-04338/2016 and A/chicken/Cameroon/16V-3771/2016.

#### Amplification and Sanger sequencing

Viral isolates were amplified for all genes and analysed for full genome sequences using the same set of primers (Supplemental Table 2). The virus from the third sample could not be isolated after two passages and was only utilized for full length sequencing of HA and NA genes due to limited sample for further analysis. Overall a total reaction volume of 50 µL contained the following reagents: 10 µL of 5× PCR buffer, 2 µL of 10 µM dNTPs, 1 µL of 10 µM forward and reverse primer, 0.25 µL RNAsin 40 U/µL, 2 µL Qiagen One-step RT–PCR enzyme and 5 µL RNA. This mixture was run using the following program: 60°C for 1 min, 42°C for 10 min, 50°C for 30 min, 95°C for 15 min, 30 cycles at (94°C for 30 s, 55°C for 30 s, 72°C for 90 s) and 72°C for 7 min. Amplicons were then sequenced by Sanger sequencing with Big Dye Terminator Reaction Mix (Applied Biosystems) on the ABI 3500XL genetic analyser. Contiguous sequences were assembled using CLC Main Workbench 5.5 (Qiagen, Hilden, Germany).

#### Phylogenetic analysis

Representative influenza gene sequences were obtained from the NCBI Influenza Virus Database (https://www.ncbi.nlm.nih.gov/genomes/FLU/Database) [43] and GISAID as accessed in November 2017. Primarily African and Asian strains were used for analysis and incomplete sequences were excluded from the analysis. Sequence alignment was executed using ClustalW in BioEdit version 7.2.5 [44]. Phylogenic relationships and tree construction for each gene were inferred by Maximum Likelihood (ML) and 500 bootstrap replicates were performed to infer the robustness of the ML trees using MEGA 6 [45]. Viruses were clustered on the basis of nucleotides. Only dominant clusters were used to infer phylogenetic relationships.

#### Visualization of changes in amino acids of the haemagglutinin (HA) protein of Cameroon isolates

Visualization of changes in amino acids on the HA of Cameroon isolates was performed using PyMOL [46] based on the structure of the HA of the clade 2.3.2.1 virus A/Hubei/1/2010 (DOI: 10.2210/pdb4kth/pdb) [47] available on the RCSB Protein Data Bank (https://www.rcsb.org/pdb/home/home.do).

#### Antigenic testing of Cameroon isolates

Haemagglutination inhibition assays (HAI) were performed on Receptor Destroying Enzyme (RDE; Denka Seiken, Tokyo, Japan)-treated samples by previously published methodology [48] using chicken, turkey, horse, and human type O RBCs against the viral isolates from Cameroon, as well as a number of other reference antigens and antiserum (Supplemental Table 3).

### Human samples

#### Ethical approval

The human investigation was conducted as part of a public health response; thus, in accordance with human subjects’ protection regulations, it was not considered to be human subjects’ research. CPC serves as the National Influenza Centre for Cameroon. Administrative authorizations were obtained from the Cameroon Ministry of Health in order to control the spread of infection. However, all participants signed an informed consent form prior to enrolment and sample collection.

#### Sample collection

Overall, necessary sample size could not be calculated as HPAIV A(H5N1) transmission to humans was unlikely and the probability of HPAIV infection in Cameroon is unknown. Therefore, as part of the outbreak response, all contact individuals identified to be present on the farms and in the markets where dead poultry was detected during the avian influenza outbreak had their blood collected for serological analyses. The longitudinal human serological study was performed during the outbreak period and involved collection of serum, nasopharyngeal (NP), and oropharyngeal (OP) swabs (swabs pooled into one tube per subject) from poultry workers and exposed individuals. Overall, samples from 633 individuals exposed to sick or dead poultry during the outbreak period were collected in 5 regions totalling 7 sites (Supplemental Table 1). NP and OP swabs were placed in viral transport medium and stored at 4°C prior to transport in a cooler. Blood specimens were collected from poultry farm and LBM workers at initial inclusion to form a baseline and two weeks after for follow-up. Blood samples were stored at 4°C and sent to the CPC within 24 h preceding collection where they were processed and 1 mL aliquots of serum and plasma were frozen at −80°C until use.

#### Testing of human samples for influenza infection by RT-qPCR

RNA was extracted from NP and OP swabs and tested for influenza A and B viruses by RT-qPCR with the CDC Influenza A/B typing assay obtained through the IRR (https://www.internationalreagentresource.org). Positive samples for influenza A virus were then tested by RT-qPCR for A(H3N2), A(H1N1)pdm09, A(H5N1), and A(H7N9) with the corresponding CDC sub-typing kits available from the IRR. All RT-qPCR analyses were run on an ABI Prism 7300 or 7500 thermocycler (Applied Biosystems, Foster City, California, USA) as described earlier. Threshold cycles (Ct values) below 37 were considered positive for all RT-qPCR analyses.

#### Serological analysis for infection with A(H5N1)

One hundred and thirty-one (131) pairs of serum samples (*n* = 262 total samples) were selected from the all of the contact patients based on: (1) the availability of matched sets of two sera collected within a two week interval; and, (2) with sufficient volume to carry out the serological assays. Following RDE treatment, serum samples were tested for HPAIV A(H5N1) antibodies using HAI as described above and confirmed with microneutralization assay (MN) using previously published methodology [49]. HAI analyses were performed using the two egg isolates from Cameroon, reference clade 2.3.2.1 A(H5N1) viruses/antigens A/common magpie/Hong Kong/5052/2007, A/environment/Cambodia/z2EP1e3W7M1/2015 and A/duck/Vietnam/NCVD-1584/2012 using horse and human RBCs. The second set of sera were first analysed by HAI for the likelihood of patients with seroconversion. Samples with detectable antibodies in the second serological draw (HAI titre ≥ 10) were then re-tested by HAI and MN assay utilizing both the first and second serum samples. Exposure to these viruses was considered “suspected” with an HAI titre ≥ 40 in the second serum sample and/or seroconversion defined as the detection of antibodies above the thresholds (fourfold increase) defined following no detection of antibodies in the serum sample from the previous period (Supplemental Table 6).
